# Change of periodontal inflammatory indicators through a 4-week weight control intervention including caloric restriction and exercise training in young Koreans: a pilot study

**DOI:** 10.1186/s12903-015-0094-7

**Published:** 2015-09-18

**Authors:** Hoo-Seob Park, Hae-Sung Nam, Hyung-Seok Seo, Soo-Jeong Hwang

**Affiliations:** Department of Preventive Medicine and Public Health, School of Medicine, Chungnam National University, Daejeon, South Korea; Department of Sports Medicine, College of Culture, Science & Technology, Konyang University, Nonsan, Chungcheongnamdo South Korea; Graduate School of Medical Science and Engineering, Korea Advanced Institute of Science and Technology (KAIST), Daejeon, South Korea; Department of Dental Hygiene, College of Medical Science, Konyang University, Daejeon, South Korea

**Keywords:** Gingival Crevicular Fluid, IL-1β, MMP-8, MMP-9, Obesity, Periodontium

## Abstract

**Background:**

Recent cross-sectional studies indicate that obesity is a risk factor for periodontal disease. Exercise training in high fat mice or rats can inhibit gingival inflammation effectively. The objective of this human intervention study was to investigate whether short-term weight control could affect periodontal indexes and serum and gingival crevicular fluid (GCF) biomarkers in young Koreans.

**Methods:**

Forty-one obese volunteers (body mass index (BMI) > 25.0) and 12 normal weight subjects (18.5 ≤ BMI ≤ 23.0) participated in a four-week weight control program to analyze the changes in anthropometric criteria, the concentrations of C-reactive protein (CRP), low-density lipoprotein (LDL), high-density lipoprotein (HDL), and triglycerides in serum, gingival index, bleeding on probing, periodontal biomarkers in GCF, and dental plaque index at the first and the 27th days.

**Results:**

The means of obesity measures decreased significantly more in the obese group (BMI 2.53 ± 0.96, waist-to-hip ratio (WHR) 4.88 ± 1.58 %, LDL 35.85 ± 21.74 mgdL^−1^) than in the normal weight group (BMI 0.78 ± 0.72, WHR 2.00 ± 0.95 %, LDL 15.58 ± 18.07 mgdL^−1^). While the obese group showed significant decreases in the biomarkers in GCF (IL-1β 58.38 ± 65.55 pgmL^−1^, MMP-8 4.19 ± 5.61 ngmL^−1^, MMP-9 3.36 ± 6.30 ngmL^−1^), the mean changes for the normal weight group (IL-1β 10.07 ± 21.08 pgmL^−1^, MMP-8 1.49 ± 4.61 ngmL^−1^, MMP-9 -1.52 ± 9.71 ngmL^−1^) were not statistically significant. Anthropometric measures and the amounts of GCF biomarkers had weak positive correlations (0.242 ≤ r ≤ 0.340), and LDL in serum correlated with MMP-8 (*r* = 0.332) and IL-1β (*r* = 0.342) in the obese group. Stepwise multiple linear regression analysis in the obese group showed that the relationship between the amount of IL-1β in GCF and predictor variables including LDL and BMI was highly significant and accounted for 19.1 % of the variance in IL-1β in GCF.

**Conclusions:**

In periodontally healthy subjects, weight control could reduce the amounts of MMP-8, MMP-9, and IL-1β in GCF of the obese subjects. Further studies with periodontally unhealthy and obese people are needed to identify the mechanism of decreases in inflammation biomarkers in GCF through weight control.

**Trial registration:**

ISRCTN86753073 (2015.08.14)

## Background

Preventive programs for periodontal disease have focused on dental plaque control since dental plaque has been regarded as the main cause of periodontal disease based on Löe’s experimental gingivitis model [37]. Some dental professionals have taken active interest in the relationship between periodontal disease and metabolic syndrome or obesity, after diabetes appeared to be correlated with the onset of periodontal disease [3, 8, 15, 21, 34, 39]. Recent cross sectional studies have indicated an association between obesity and periodontal disease. Body mass index (BMI), waist-hip ratio (WHC), or waist circumference (WC) were significantly associated with periodontal disease or the number of remaining teeth [11, 13, 29, 33, 35, 43, 50, 52, 58, 59]. Although positive correlations between periodontal disease and obesity have been shown across diverse populations in cross-sectional studies [10], the causal relationship in human studies has been unclear in a few systematic reviews [36, 57] and prospective studies [14, 25, 41, 54].

However, the diet-induced obese mouse model best serves research studies relevant to the cause-and-effect relation between periodontal health and obesity [3]. Obese rat studies have shown that obesity could contribute to the severity of periodontal disease [46], alveolar bone loss [12] and spontaneous periodontal disease [9]. Exercise training in high-fat diet mice and rats could effectively suppress oxidative stress in gingival tissue [4] and markedly inhibit tumor necrosis factor (TNF)-α in adipose tissue [27]. Caloric restriction had a significant decrease effect on inflammatory mediators in gingival crevicular fluid (GCF) [17].

Clinical periodontal indexes, including periodontal probing depth, gingival index, and plaque index, have relatively low reproducibility and reliability. In place of these clinical indicators, some enzymes and pro-inflammatory cytokines in GCF have been used as indicators of periodontal inflammation in studies to identify small changes related to periodontal health. Matrix metalloproteinase (MMP)-8 and MMP-9 were considered as the most competent collagenase and gelatinase to initiate type I collagen and extracellular matrix degradation in periodontal tissue [20, 23, 38, 56]. Pro-inflammatory cytokines, such as Interleukin (IL)-1β, inhibit collagen synthesis and contribute to collagen degradation [6, 48].

This human study tested whether weight-control intervention including caloric restriction and exercise training in the obese could change the periodontal biomarkers within 4 weeks as a pilot study.

## Methods

### Study design and subjects

This study was designed as a pre-post study on the effects of a 4-week weight control program on a few biomarkers in GCF. The study was approved by the Ethical Committee of Konyang University Hospital.

The concentration of MMP-8 in GCF was selected as the main outcome variable to confirm changes in periodontal health on the basis of previous studies [48, 55, 56] that suggested MMP-8 was a key mediators of gingival tissue destruction. We fixed 6.3 ng/ml of MMP-8 as a clinical allowable error on the basis of preliminary research, with a significance level of *p* = 0.05 and 80 % level of power; forty-eight subjects were determined as the sample size for a paired *T*-test.

Forty-nine obese individuals in their twenties with BMI of >25 kgm^−2^ (Asia-Pacific obesity criteria) and 13 camp trainers of the same age with 18.5 ≤ BMI ≤ 23 kgm^−2^ participated in the 4-week weight-control program at Konyang University in Korea in 2011. Individuals were excluded if they presented any of following-: (1) systemic disease exclusive of obesity, (2) use of steroidal or non-steroidal anti-inflammatory drugs or antibiotics in the last three months or during the program, (3) use of mouthwash in the last three months or during the program, (4) need of dental or medical treatment during the program, (5) fewer than twenty-four teeth, (6) sites with probing periodontal pocket depth (PD) > 3.5 mm (due to the ethical problem of prohibition of periodontal treatment during the camp), and (7) self-directed dropout during the course of the weight-control program. Eight obese subjects and one normal-weight subject dropped out of the camp by their own choice.

**Fig. 1:**
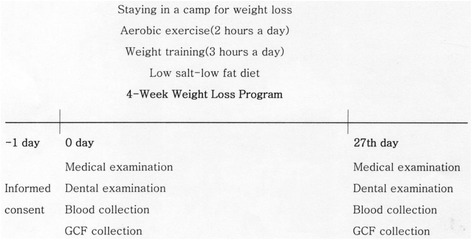
Flow of 4-week weight controlled intervention

All subjects provided signed informed consent as required by Konyang University Hospital Institutional Review Board. As shown in Fig. 1 and Table 1, they stayed in the camp under surveillance for two hours of aerobic exercise, three hours of weight training, and a low salt-low fat diet (≤1300 kcal/day). They were not allowed to have any private foods or drinks except water. We examined the dental plaque index at the baseline and final state to serve as a proxy for the maintenance of the subjects’ habitual oral health behavior. We made no attempt to change the subjects’ toothbrushing method or frequency so as not to affect gingival inflammation during the program. Smoking was not prohibited to maintain the other conditions as confounding factors except weight control.

**Table 1:** Partial examples of dietary tables in the weight control camp

	The 1st day			The 2nd day			The 3rd day		
	Food	Weight(g)	Kcal	Food	Weight(g)	Kcal	Food	Weight(g)	Kcal
Breakfast	Boiled potato	130	109	Breads with mixed grain	49	130	Curried rice with chicken	200	301
	Banana	100	100	Boiled chicken breasts	100	165	Cucumber	200	38
	Low-fat milk	200	102	Tomatoes	100	12			
				Yoplait	85	75			
Lunch	Brown rice	105	160	Rice with beans	150	177	Rice with beans	105	177
	Bean paste soup with tofu	175	50	Soup with egg	200	45	Boiled pork	50	192
	Grilled mackerel	50	106	Smoked duck	100	154	Lettuce	15	3
	Bean sprouts	35	25	Roasted mushroom	100	40	White radish	100	40
	Cucumber	100	19	Boiled egg	50	72.5	Watermelon	300	93
	Boiled egg	50	72.5				Boiled egg	50	72.5
Dinner	Rice with beans	105	177	Rice with beans	105	177	Steamed sweet pumpkin	200	58
	Sea mustard soup	250	90	Soup with bean sprouts	170	25	Almond	100	45
	Roasted tofu	50	60	Roasted tofu	50	60	Boiled chicken breasts	40	50
	Spinach	50	40	Mung bean jelly	80	100	Tomato	100	12
	Lettuce	50	70	Banana	100	93	Banana	100	93
	Banana	100	93						

The mean ages of the subjects were 22.79 ± 2.37 (year) in the obese group, and 22.23 ± 2.42 (year) in the normal weight group. Female subjects compromised 65.9 % (27 subjects) of the obese group and 50.0 % (6 subjects) of the normal weight group. The mean BMI at the base line was 28.44 ± 2.61 in the obese group, and 22.20 ± 0.75 in the control group. There were 35 non-smokers (85.4 %) in the obese group and 8 (66.7 %) in the control group.

### Clinical examinations and sample collections

Anthropometric data, oral examination data, GCF, and whole blood samples were collected from 9 A.M. to 11 A.M. at the baseline and at the 27th day (Fig. 1). Weight, BMI, body fat mass, percent body fat, WHR and WC were measured using the body composition analyzer with direct segmental multi-frequency - bioelectric impedance analysis (InBody, InBody Co., Seoul, Korea). Oral examination consisted of dental plaque index (Sum of Turesky modification of the Quingley-Hein index) after ordinary toothbrushing, gingival index (Sum of Löe-Silness gingival index), sites of bleeding on probing (BOP), and PD with WHO periodontal probe by one dentist who was calibrated by the Korean National Oral Health Survey. Each sum of clinical indicators was chosen instead of the mean to show the slight differences of plaque index and gingival index in four weeks; all subjects had the same numbers of teeth and comparatively healthy condition of periodontium.

Blood samples were collected before breakfast after the subjects had fasted for more than 8 h, and the samples were immediately transferred to the department of laboratory medicine, Konyang University Hospital at 4 °C. On the same day, concentration of C-reactive protein (CRP) as a systemic inflammation indicator, concentrations of low-density lipoprotein cholesterol (LDL), triglycerides, and high-density lipoprotein cholesterol (HDL) as obesity indicators in the blood samples were measured. GCF samples were collected at the 5 interproximal areas of maxillary anterior teeth with #25 sterilized paper points using the intracrevicular method “superficial” [19] for 1 min after removing dental plaque. The GCF samples of each subject were stored in a sterile 1 ml Eppendorf tube and frozen at -70 °C until processing.

The GCF samples were eluted into 1 mL phosphated buffered saline for 60 min at the room temperature. Enzyme-linked immunosorbent assay (ELISA) was used to measure the amounts of IL-1β, MMP-8, MMP-9 in GCF using the Human IL-1β ELISA Kit (Promokine, Heidelberg, Germany), Quantikine human MMP-8 (R&D systems, Minneapolis, USA) and Quantikine human MMP-9 (R&D systems, Minneapolis, USA). The representative values of data in the blood and GCF samples were calculated as means in triplicate.

### Statistical analysis

The null hypothesis was that there would be no significant clinical and biochemical changes of periodontal indicators during the 4-week weight control program. Parametric statistics of the data were distributed normally except the gingival index for the normal weight group. A paired *t*-test was utilized to determine whether there were significant differences between the pre- and post-program values. The *t*-test was used to identify differences between the data of obese and normal weight subjects. Correlation analysis using Pearson’s coefficients among periodontal indicators and obesity indicators was applied to all data at the baseline and the 27^th^ day. Stepwise multiple linear regression analysis of the obese group’s data was used to examine the relationships between the amounts of IL-1β in GCF and obesity indicators (LDL and BMI) because IL-1β showed slightly stronger positive correlations with obesity indicators than MMP-8. The level of statistical significance was set at α = 0.05. All statistical analyses were performed with the IBM SPSS 20.0 (IBM Co., Armonk, NY, USA).

## Results

All 41 obese subjects succeeded in losing weight during the 4-week weight control program. Tables 2 and 3 summarize the differences between the obese and normal weight groups in terms of obesity indicators and periodontal indicators. Obesity measures of BMI, WHR, WC and concentrations of LDL in serum decreased significantly, although the concentration of HDL changed insignificantly, and triglycerides in serum increased contrary to expectations. The concentration of CRP in serum was measured to confirm systemic inflammation, and it did not show a significant change during the program. The macroscopic data regarding periodontal health, including gingival index (Sum of Löe-Silness gingival index) and sites of BOP, did not show significant decrease for the large standard deviation. Nevertheless, the amounts of IL-1β, MMP-8 and MMP-9 in the GCF of the obese group as gingival inflammatory markers declined 60.2, 58.6, and 35.8 % respectively. The normal weight group showed insignificant decreases in the amounts of periodontal biomarkers in GCF during the weight control program. The obese group showed greater differences in WC, WHR, BMI, LDL, IL-1β, and MMP-9 in comparison with the normal weight group.

**Table 2:** Comparison of pre-post differences in obesity indicators between the obese group and normal weight group

	Obese^a^ (*n* = 41)				Normal weight^a^ (*n* = 12)				
	Baseline	27th day	Difference	P*	Baseline	27th day	Difference	P*	P**
Obesity index									
WC(cm)	97.62 ± 17.17	86.65 ± 6.34	10.91 ± 14.41	<0.001	84.58 ± 3.92	83.26 ± 3.26	1.33 ± 1.30	0.005	<0.001
WHR(%)	90.15 ± 4.22	85.29 ± 3.62	4.88 ± 1.58	<0.001	82.17 ± 2.62	80.17 ± 2.17	2.00 ± 0.95	<0.001	0.027
BMI(kgm^−2^)	28.44 ± 2.61	25.90 ± 2.21	2.53 ± 0.96	<0.001	22.20 ± 0.75	21.42 ± 0.61	0.78 ± 0.72	0.003	<0.001
Cholesterol and inflammatory indicators in serum									
CRP(mgdL^−1^)	0.23 ± 0.23	0.33 ± 1.37	−0.10 ± 1.38	0.652	0.13 ± 0.21	0.06 ± 0.08	0.06 ± 0.20	0.299	0.691
LDL(mgdL^−1^)	123.20 ± 24.57	87.35 ± 17.80	35.85 ± 21.74	<0.001	98.75 ± 21.69	82.17 ± 15.26	15.58 ± 18.07	0.009	0.007
Triglycerides(mgdL^−1^)	57.43 ± 13.88	72.23 ± 13.16	−13.80 ± 13.56	<0.001	43.70 ± 11.21	60.91 ± 16.67	−17.21 ± 13.84	0.001	0.451
HDL(mgdL^−1^)	53.50 ± 9.14	53.57 ± 10.07	−0.07 ± 9.17	0.964	59.96 ± 10.17	61.27 ± 11.48	−1.31 ± 8.16	0.590	0.675

* paired *T*-test between the data of baseline and that of 27th day in each group

** *T*-test between the difference of the obese and that of the normal weight

^a^Mean ± s.d.

**Table 3:** Comparison of pre-post differences in periodontal health indicators between the obese group and normal weight group

	Obese^a^ (*n* = 41)				Normal weight^a^ (*n* = 12)				
	Baseline	27th day	Difference	P*	Baseline	27th day	Difference	P*	P**
Dental index									
Dental plaque index	51.68 ± 17.85	74.61 ± 33.03	−22.93 ± 33.71	<0.001	42.33 ± 27.32	58.17 ± 32.77	−15.83 ± 21.99	0.030	0.496
Gingival index	22.95 ± 55.20	19.39 ± 29.85	3.56 ± 43.71	.0.605	43.75 ± 95.71	30.58 ± 62.59	13.17 ± 38.72	0.264	0.496
Sites of BOP	10.12 ± 27.64	5.85 ± 12.94	4.27 ± 22.63	0.234	19.50 ± 48.48	11.08 ± 32.20	8.41 ± 17.89	0.131	0.563
Periodontal inflammatory indicators in gingival crevicular fluid									
IL-1β(pgmL^−1^)	96.89 ± 62.53	38.51 ± 37.72	58.38 ± 65.55	<0.001	66.61 ± 58.46	56.54 ± 50.16	10.07 ± 21.08	0.642	0.033
MMP-8(ngmL^−1^)	7.24 ± 6.02	3.05 ± 3.46	4.19 ± 5.61	<0.001	5.16 ± 4.07	3.66 ± 2.60	1.49 ± 4.61	0.286	0.134
MMP-9(ngmL^−1^)	9.46 ± 4.95	6.11 ± 5.17	3.36 ± 6.30	0.002	7.67 ± 5.53	9.20 ± 8.04	−1.52 ± 9.71	0.597	0.043

* paired *T*-test between the data of baseline and that of 27th day in each group

** *T*-test between the difference of the obese and that of the normal weight

Dental plaque index: Sum of Turesky modification of the Quingley-Hein index

Gingival index: Sum of Löe-Silness gingival index

^a^Mean ± s.d.

Tables 4 and 5 demonstrate the correlations between obesity and gingival health indicators in the obese group and the normal weight group, respectively. The concentrations of IL-1β (*r* = 0.342) and MMP-8 (*r* = 0.332) in GCF showed weak positive correlations with LDL values in serum in the obese group. IL-1β in GCF indicators showed weak positive correlations with BMI (*r* = 0.340) and WHR (*r* = 0.270) among the anthropometric data. MMP-8 in GCF indicators showed weak positive correlation with WC (*r* = 0.254), WHR (*r* = 0.242) and BMI (*r* = 0.234). The numbers of sites of BOP showed weak positive correlations with IL-1β (*r* = 0.220), MMP-8 (*r* = 0.293), and LDL (*r* = 0.233), but they showed no correlations with anthropometric data. Moderate to strong positive correlations were seen in the interrelations among GCF biomarkers (0.568 ≤ r ≤ 0.772) and anthropometric data (0.640 ≤ r ≤ 0.924), respectively. Table 5 shows that the normal weight group showed weaker correlations between obesity and gingival health indicators in comparison with Table 4. However, moderate positive correlations were seen in the relationships of IL-1β and WC (*r* = 0.474), MMP-8 and WHR (*r* = 0.438), the numbers of sites of BOP and IL-1β (*r* = 0.421), the numbers of sites of BOP and LDL (*r* = 0.455), and the numbers of sites of BOP and HDL (*r* = -0.621).

**Table 4:** Pearson coefficients for obesity and gingival health indicators in the obese group at the 1st day and 27th day of weight control

	WC	WHR	BMI	LDL	Triglycerides	HDL	BOP	IL-1β	MMP-8
WHR	0.640**								
BMI	0.664**	0.924**							
LDL	0.217	0.264*	0.214						
Triglycerides	0.006	0.064	0.059	−0.147					
HDL	−0.173	−0.263*	−0.240*	0.078	−0.179				
BOP	−0.103	−0.173	−0.114	0.233*	−0.020	0.119			
IL-1β	0.200	0.270*	0.340**	0.342**	−0.274*	0.073	0.220*		
MMP-8	0.254*	0.242*	0.234*	0.332**	−0.061	0.114	0.293**	0.596**	
MMP-9	0.159	0.144	0.137	0.206	−0.118	0.013	0.181	0.568**	0.772**

*: *p* < 0.05, **: *p* < 0.01

**Table 5:** Pearson coefficients for obesity and gingival health indicators in the normal weight group at the 1st day and 27th day of weight control

	WC	WHR	BMI	LDL	Triglycerides	HDL	BOP	IL-1β	MMP-8
WHR	0.833**								
BMI	0.575*	0.789**							
LDL	0.019	0.076	0.284						
Triglycerides	−0.108	−0.271	−0.258	0.089					
HDL	−0.114	−0.293	−0.154	−0.154	−0.194				
BOP	0.396	0.337	0.185	0.455*	0.377	−0.621**			
IL-1β	0.474*	0.404	0.156	−0.115	0.076	−0.355	0.421*		
MMP-8	0.350	0.438*	0.212	−0.277	−0.282	−0308	0.036	0.707**	
MMP-9	0.271	0.236	−0.005	−0.422*	−0.153	0.037	−0.008	0.668**	0.713*

*: *p* < 0.05, **: *p* < 0.01

Stepwise multiple linear regression analysis in the obese group was used to examine the relationship between the outcome variable, the amount of IL-1β in GCF, and predictor variables, including LDL and BMI (Table 6). This model was highly significant (*p* < 0.001) and accounted for 19.1 % of the variance in IL-1β in GCF (r^2^ = 0.191, adjusted r^2^ = 0.170).

**Table 6:** Multiple linear regression analysis as predictors of the amount of IL-1β in GCF in the obese group

	Un-standardized coefficient	SE	Standardized coefficient	*p*
intercept	−157.792	61.089		0.012
LDL	0.593	0.219	0.283	0.008
BMI	6.026	2.260	0.278	0.009

*r* ^2^ = 0.191, adjusted *r* ^2^ = 0.170. Overall *p*-value < 0.001

## Discussion

Obesity might be both an indirect risk factor because it affects glycemic control, and a direct risk factor because the secretion of pro-inflammatory agents by adipose tissue modifies periodontal reactions to the plaque biofilm [7, 15, 28, 30, 34, 39, 42]. In addition, obesity could interfere with the ability of the immune system to appropriately respond to *Porphyromonas gingivalis* infection in mice with diet-induced obesity through altered function of macrophages [2, 60]. Adipose tissue secretes several cytokines and hormones that are involved in inflammatory processes, suggesting that obesity and periodontitis might share the same pathophysiologic pathway [15, 47].

Previous research regarding caloric restriction and exercise showed improvement of gingival condition as well as systemic condition. Some animal studies have shown that obesity prevention by exercise training might significantly suppress gingival inflammation [4, 17]. Moderate daily exercise with dietary control could restore the impaired cytokine responses in diet-induced obese mice and improve the resolution of *Porphyromonas gingivalis*-induced periodontitis [61]. Also, a low physical activity level and a poor diet in human were significantly associated with increased odds of periodontal disease [5].

We hypothesized that weight control intervention in obese subjects could decrease the concentrations of periodontal biomarkers through a decrease of systemic inflammation. We chose CRP [22, 24, 45, 51] in serum as a representative systemic inflammation marker, MMP-8, MMP-9 [16, 21, 31] in GCF as those of gingival tissue destruction markers, and IL-1β [26, 31] in GCF as that of proinflammatory cytokines in gingival tissue. This study showed that exercise and diet control led to a decline in the concentrations of MMP-8, MMP-9, and IL-1β in GCF in the obese group, notwithstanding the 45.2 % increase in dental plaque burden and no significant change in clinical periodontal parameters (Table 3). On the other hand, Kondo et al. [32] showed that a high-fiber, low-fat diet improved the clinical periodontal disease markers of PD, clinical attachment and BOP.

Buduneli et al. [8] found that obesity did not seem to have a significant effect on clinical periodontal parameters but that it had many correlations with systemic circulating inflammatory molecules. On the other hand, Morel et al. [40] showed that the short-term adoption of a very low-calorie diet in obese females made the total cholesterol, HDL, LDL, and triglycerides decrease, but changes in TNF-alpha and, IL-6 levels did not reach statistical significance. We could not find a direct link between systemic inflammation and periodontal biomarkers in GCF periodontal health because the systemic inflammation marker, CRP in serum, did not change (Table 2) and had no correlation with other variables (data not shown). In addition, it is not certain whether changes in periodontal biomarkers in GCF resulted from a decrease in systemic inflammation or local inflammation because the same inflammation markers in GCF and serum were not analyzed, and GCF is a transudate from healthy gingival tissue or an exudate from inflammatory gingival tissue including blood filtrate.

In the study of relationship between cholesterol and periodontal health, a significant association was seen between LDL, triglyceride, and cholesterol and the severity of periodontitis [44]. Obese subjects with a high serum triglyceride or LDL level and/or a low HDL-cholesterol level could be at higher risk of periodontal infection [22, 53]. Inversely, our study showed that triglycerides had weak negative correlation with IL-1β in GCF, although LDL had weak positive correlations with IL-1β and MMP-8 in the GCF of obese subjects (Table 4). A low-fat, high-carbohydrate diet can increase triglycerides in serum, yet Table 1 shows that the portion of carbohydrates in the daily diet was less than 50 %. The peculiar increase in the concentrations of triglycerides in serum after this low salt-low fat diet supported that the findings of a previous study that showed that sodium reduction resulted in a 2.5 % increase in cholesterol and a 7 % increase in triglyceride [18].

There were several limitations to our study to prove whether exercise and diet modification as a treatment for obesity could improve periodontal condition. The sex ratios and smoker ratios of the obese group and the normal weight group were different, although the GCF of the female subjects could be influenced by periodic variation of sex hormone levels [1] and smoking was a significant factor in the development and progression of periodontal disease [49]. We tried to use more objective variables, such as IL-1β, MMP-8, and MMP-9 in GCF, rather than clinical indicators. However, fixed sites in the upper anterior teeth were chosen for collection of GCF irrespective of the periodontal condition of each site, and the amounts of GCF at each site could not be measured. Besides, our obese subjects in their twenties had relatively healthy periodontium condition and had no problems in CRP, LDL, triglycerides, and HDL except anthropometric data. An obese group with periodontal pockets and abnormal concentrations of cholesterol might show different results.

Nonetheless, this study was meaningful as a human pilot study to show that obesity control by exercise and diet control could reduce gingival inflammatory biomarkers without periodontal intervention. Not only clinical indexes, but also biomarkers in blood and GCF were analyzed. We found that obesity and periodontal indicators showed weak positive correlations, and LDL and BMI accounted for 19.1 % of the variance in IL-1β in the GCF of obese subjects (Table 6). We suggest that a weight control study with an obese group with periodontal inflammation could provide substantial evidence that weight control can improve periodontal condition.

## Conclusion

Within its limits, this study demonstrated that weight control including exercise and diet restriction for 4 weeks could reduce the amounts of periodontal biomarkers in GCF as well as obesity, without periodontal intervention. BMI, WC, and WHR showed weak positive correlations with IL-1β and MMP-8 in GCF, and LDL in serum and BMI accounted for 19.1 % of the variance in IL-1β in GCF.

## Abbreviations

BMI: Body mass index
BOP: Bleeding on probing
CRP: C-reactive protein
ELISA: Enzyme-linked immunosorbent assay
GCF: Gingival crevicular fluid
GI: Gingival index (Sum of Löe-Silness gingival index)
HDL: High-density lipoprotein
IL: Interleukin
LDL: Low-density lipoprotein
MMP: Matrix metalloproteinase
PD: Probing periodontal pocket depth
SE: Standard error
WC: Waist circumference
WHR: Waist-to-hip ratio
